# Human IgG responses to the *Aedes albopictus* 34k2 salivary protein: analyses in Réunion Island and Bolivia confirm its suitability as marker of host exposure to the tiger mosquito

**DOI:** 10.1186/s13071-022-05383-8

**Published:** 2022-07-20

**Authors:** Sara Buezo Montero, Paolo Gabrieli, Anne Poinsignon, Bi Zamble Hubert Zamble, Fabrizio Lombardo, Franck Remoue, Bruno Arcà

**Affiliations:** 1 Department of Public Health and Infectious Diseases, Division of Parasitology, Sapienza University of Rome, Piazzale Aldo Moro 5, 00185 Rome, Italy; 2 Department of Biosciences, University of Milan, Via Celoria 26, 20133 Milan, Italy; 3 MIVEGEC, University of Montpellier, IRD, CNRS, 34000 Montpellier, France; 4 Present Address: Institute of Tropical Medicine, University of Tübingen, Wilhelmstrasse 27, 72074 Tübingen, Germany

**Keywords:** *Aedes albopictus*, *Aedes aegypti*, 34k2 salivary protein, Host exposure, Serological marker, Vector control, Human-vector contact

## Abstract

**Background:**

The rapid worldwide spreading of *Aedes aegypti* and *Aedes albopictus* is expanding the risk of arboviral diseases transmission, pointing out the urgent need to improve monitoring and control of mosquito vector populations. Assessment of human-vector contact, currently estimated by classical entomological methods, is crucial to guide planning and implementation of control measures and evaluate transmission risk. Antibody responses to mosquito genus-specific salivary proteins are emerging as a convenient complementary tool for assessing host exposure to vectors. We previously showed that IgG responses to the *Ae. albopictus* 34k2 salivary protein (al34k2) allow detection of seasonal and geographic variation of human exposure to the tiger mosquito in two temperate areas of Northeast Italy. The main aim of this study was to confirm and extend these promising findings to tropical areas with ongoing arboviral transmission.

**Methods:**

IgG responses to al34k2 and to the *Ae. aegypti* orthologous protein ae34k2 were measured by ELISA in cohorts of subjects only exposed to *Ae. albopictus* (Réunion Island), only exposed to *Ae. aegypti* (Bolivia) or unexposed to both these vectors (North of France).

**Results and conclusion:**

Anti-al34k2 IgG levels were significantly higher in sera of individuals from Réunion Island than in unexposed controls, indicating that al34k2 may be a convenient and reliable proxy for whole saliva or salivary gland extracts as an indicator of human exposure to *Ae. albopictus*. Bolivian subjects, exposed to bites of *Ae. aegypti*, carried in their sera IgG recognizing the *Ae. albopictus* al34k2 protein, suggesting that this salivary antigen can also detect, even though with low sensitivity, human exposure to *Ae. aegypti*. On the contrary, due to the high background observed in unexposed controls, the recombinant ae34k2 appeared not suitable for the evaluation of human exposure to *Aedes* mosquitoes. Overall, this study confirmed the suitability of anti-al34k2 IgG responses as a specific biomarker of human exposure to *Ae. albopictus* and, to a certain extent, to *Ae. aegypti*. Immunoassays based on al34k2 are expected to be especially effective in areas where *Ae. albopictus* is the main arboviral vector but may also be useful in areas where *Ae. albopictus* and *Ae. aegypti* coexist.

**Graphical Abstract:**

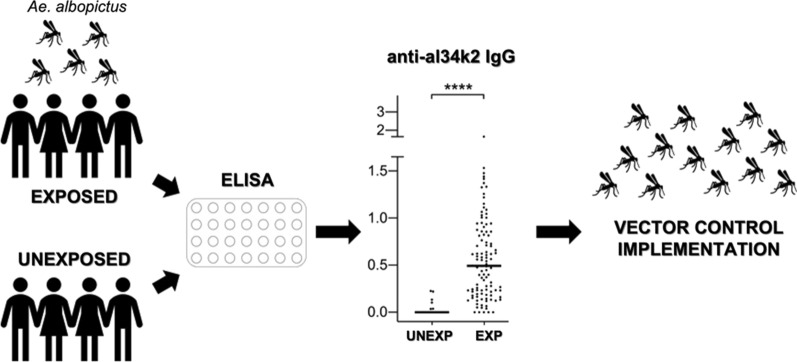

## Background

Arboviral diseases transmitted by mosquitoes (such as dengue, chikungunya, Zika, West Nile or yellow fever) are a serious concern for public health, and > 50% of the world’s population is considered at risk of contracting at least one of these arbovirosis [1]. Except for West Nile virus, commonly transmitted by *Culex* mosquitoes, all other arboviruses mentioned above are transferred to humans by *Aedes* species. The major vector is certainly the yellow fever mosquito *Aedes aegypti*, but the Asian tiger mosquito *Aedes albopictus* can play a relevant vectorial role, especially in areas where *Ae. aegypti* is absent or present at low density [2–4]. The geographical distribution of these two species increased impressively during the past decades [5], extending the risk of *Aedes*-borne arboviral diseases to temperate areas [6], as well illustrated by the repeated cases of chikungunya (CHIKV), dengue (DENV) and Zika virus (ZIKAV) transmission in continental Europe [7–9]. No specific anti-viral drugs are currently available against these arboviral diseases. A safe and effective vaccine against yellow fever has been available since the end of 1930s, and a dengue vaccine was recently licensed; nevertheless, yellow fever is still endemic in several tropical countries [10], and the use of the dengue vaccine has relevant limitations [11, 12]. For these reasons, the containment of these arboviral diseases relies almost exclusively on vector monitoring and control and on reducing the contact between the human host and the mosquito vector. Insecticides are still the most effective weapon for vector control. However, the spreading of resistance among mosquito vector populations points to the need for a careful management of insecticides [13] and for the development of novel approaches [14, 15]. In this respect, very promising results have been obtained by *Wolbachia*-infected *Ae. aegypti* for the containment of dengue [16, 17].

The evaluation of human-vector contact is of fundamental importance to monitor *Aedes* populations, to assess the risk of arboviral transmission and for planning/implementation of vector control measures. This is usually carried out by classical entomological methods [18], which, however, have some intrinsic limitations since they can be labor-/time-consuming, may raise ethical concerns (human landing catches) and only provide indirect estimations at the community level. The use of IgG responses to mosquito salivary proteins emerged as a convenient complementary or alternative tool to evaluate human-vector contact and assess the risk of mosquito-borne diseases. First demonstrations used mosquito saliva or salivary gland extracts (SGE) [19–22]; later on, the identification of *Anopheles*- and *Aedes*-specific salivary proteins [23, 24] paved the way for the development of immunoassays based on single genus-specific salivary proteins, with improvement of reproducibility and minimization of potential cross-reactivities. IgG responses to the *Anopheles gambiae* salivary protein gSG6, or the derived gSG6-P1 peptide, were shown to be reliable indicators of human exposure to malaria vectors in several different epidemiological settings [25–32], and a few additional anopheline salivary proteins have been tested or proposed as candidate markers [33–35]. These encouraging results stimulated the development of similar tools for *Aedes* vectors [36, 37], and promising indications have been obtained with the Nterm-34 kDa peptide, which is designed on the culicine-specific 34k1 salivary protein from *Ae. aegypti* and could detect human exposure to *Ae. aegypti* and possibly to *Ae. albopictus* [38–42].

We focused our attention on another member of the culicine-specific 34-kDa family, the 34k2 salivary proteins of *Ae. albopictus* and *Ae. aegypti*, which have limited amino acid identity to 34k1 (32–33%) and to orthologues from Culex species (< 30%) [43, 44]. Using a murine model, we initially showed that recombinant 34k2 proteins from *Ae. albopictus* (al34k2) and *Ae. aegypti* (ae34k2) were immunogenic to mice; intriguingly, sera of mice experimentally immunized to *Ae. albopictus* saliva did not carry IgG antibodies recognizing the ae34k2 protein, and vice versa, indicating the absence of immune cross-reactivity [45]. Moreover, using sera collected from adult healthy donors in two *Ae. albopictus* colonized areas of Northeast Italy (Padova and Belluno), we could show that anti-al34k2 IgG responses appeared appropriate to detect spatial and seasonal variation of human exposure to *Ae. albopictus* [46]. The aim of this study was to verify and validate, in a different epidemiological setting, the suitability of al34k2 for the assessment of human exposure to *Ae. albopictus* and to get additional insights into usefulness and suitability of 34k2 salivary proteins as universal or species-specific markers of human exposure to *Aedes* mosquitoes. To this end, we analyzed IgG responses to the al34k2 and ae34k2 salivary proteins in individuals from tropical areas with ongoing arboviral transmission. Specifically, in populations from Réunion Island, where individuals were naturally exposed only to *Ae. albopictus*, and from an area of Bolivia where only *Ae. aegypti* was present.

## Methods

### Ethics statement

All studies followed ethical principles as stipulated in the Edinburgh revision of the Helsinki Declaration. The studies in Réunion Island and the North of France were approved by a French Ethics Committee (the Sud Ouest, Outre Mer Ethics Committee, 25/ 02/2009) and authorized by the French Drug Agency (AFFSAPS, Ministry of Health; 12/01/2009). The study in Bolivia was approved by the Bolivian Committee of Bioethics (September 2006) and the Institut de Recherche pour le Dévelopement (IRD) "*Comité Consultatif de Déontologie et d’Ethique*" (July 2006). Written informed consent was obtained from every subject.

### Study population

Sera analyzed in this study represent subsets of samples collected from adult individuals in Réunion Island and in Bolivia and used in previous investigations to measure IgG responses to *Ae. albopictus* and *Ae. aegypti* SGEs, respectively [20, 21]. The Bolivian subset (*n* = 115; age range 18–78, mean age 35.3 years old) was selected, according to availability and age > 18 years old, from a large group of sera collected from April to May 2007 in an urban area of Santa Cruz de la Sierra [21]. In this area *Ae. aegypti* was responsible for several dengue outbreaks in the years preceding the survey, and individuals were exposed to *Ae. aegypti* (but no to *Ae. albopictus*). Sera from Réunion Island (*n* = 108; age range 18–30, mean age 24.0 years old) were collected in the city of Le Tampon during the seasonal peak of *Ae. albopictus* (May–June 2009). *Aedes aegypti* populations are present in specific locations on Réunion Island [47], but they are absent in the study area where *Ae. albopictus* is widely spread and was responsible for the large chikungunya and dengue outbreaks in 2005 and 2018, respectively [3, 4]. Sera collected from healthy adult donors from a region in North of France, free of both *Ae. albopictus* and *Ae. aegypti*, were used as unexposed control groups: note that the cohorts C (*n* = 20) used in this study and cohort C1 (*n* = 18), previously employed by Doucoure and collaborators to measure IgG responses to SGE in Réunion Island and Bolivia [20, 21], were composed of different individuals.

### Evaluation of human IgG Ab levels

The recombinant 34k2 salivary proteins from *Ae. albopictus* (al34k2, AAV90690) and *Ae. aegypti* (ae34k2, AAL76018) were expressed in *E. coli* and purified by a double step of immobilized metal ion affinity chromatography (IMAC) followed by gel filtration as previously described [45]. Enzyme-linked immunosorbent assays (ELISA) were performed as previously described [45, 46] with very few modifications. Briefly, flat-bottom 96-well plates (Nunc MaxiSorp, 442404) were coated for 3 h at room temperature (RT) with 50 µl of recombinant protein (5 µg/ml) diluted in coating buffer (15 mM Na_2_CO_3_, 35 mM NaHCO_3_, 3 mM NaN_3_, pH 9.6). After washings, wells were incubated (3 h, RT) with blocking buffer [150 μl, 1% w/v skimmed dry milk in PBST (1 × PBS, 0.05% Tween 20)], washed again and then incubated overnight at 4 °C with 50 μl of serum diluted 1:25 in blocking buffer. After washings, plates were incubated (3 h, RT) with 100 μl of polyclonal rabbit anti-human IgG/horseradish peroxidase (HRP) antibody (Dako P0214) diluted 1:5000. After washing, the colorimetric development was carried out (15 min, 25 °C in the dark) with 100 μl of o-phenylenediamine dihydrochloride (OPD, Sigma P8287). The reaction was terminated adding 25 μl of 2 M H_2_SO, and the optical density at 492 nm (OD_492_) was determined using a Biotek Synergy HT microplate reader. Washings always consisted of a first washing with PBS-T followed by three additional washings with distilled water.

### Normalization and data analysis

All samples were analyzed in duplicate with the antigen and once with no antigen for background subtraction. IgG levels, expressed as OD values at 492 nm, were calculated for each sample as the mean OD value with antigen minus the OD value without antigen. Samples with coefficient of variation between duplicates > 20% were retested or not included in the analysis. To control for intra- and inter-assay variation, each plate included negative controls and a standard curve made by three-fold dilution series (1:3–1:6561) of a pool of sera identified as hyperimmune to *Ae. albopictus* saliva in a previous study [46]. OD values were normalized using titration curves and the Excel software with a three variable sigmoid model and the Solver add-in application as previously described [48]. The mean OD values of unexposed controls plus 3xSD were used as cut-off values for seropositivity, and they were as follows: al34k2, 0.257; ae34k2, 1.017; alSGE, 0.269; aeSGE, 0.160. Prevalence was expressed as percentage ± 95% confidence interval. The Mann-Whitney *U* test and the Wilcoxon matched-pairs signed rank test were used for the pairwise comparisons among independent and paired groups, respectively. Proportions were compared by the Fisher’s exact test. Graph preparation and statistical analyses were performed using the Prism 8.0 GraphPad Software (San Diego, CA).

## Results and discussion

### IgG responses to al34k2 in individuals from Réunion Island naturally exposed to *Aedes albopictus*

Using sera collected in two areas colonized by *Ae. albopictus* in Northeast Italy, we previously found that IgG responses to the al34k2 salivary protein may be suitable as a marker to evaluate both geographical and temporal variation of human exposure to the tiger mosquito [46]. To reinforce these promising indications and provide a validation in an epidemiological setting with ongoing arboviral transmission, we determined IgG responses to al34k2 in a group of naturally exposed individuals from Réunion Island. This cohort appeared especially appropriate since IgG responses to *Ae. albopictus* salivary gland extracts (alSGE) had been previously measured in these same individuals by Doucoure and colleagues [20]. Moreover, in the area under study *Ae. albopictus* is the only *Aedes* species known to bite humans, whereas *Ae. aegypti* is absent. In these conditions IgG responses to al34k2 can be interpreted with minimal confounding effects due to exposure of the resident population to other *Aedes* species.

**Fig. 1:**
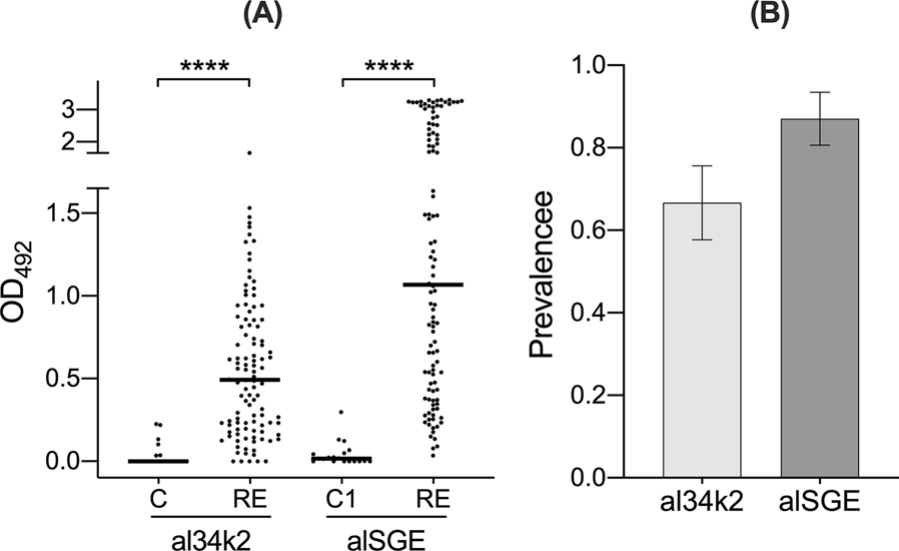
IgG responses to al34k2 in individuals from Réunion Island and unexposed controls. **A** Scatter plot of IgG responses to al34k2 in subjects from Réunion Island (RE, *n* = 108) and in unexposed controls (C *n* = 20). IgG responses to alSGE in the same individuals (RE, *n* = 108) and in unexposed controls (C1 *n* = 18), as previously determined [20], are shown for visual comparison. Horizontal bars indicate median values. al34k2, *Ae. albopictus* 34k2 recombinant protein; alSGE, *Ae. albopictus* salivary gland extracts. IgG responses are expressed as OD values. The non-parametric Mann-Whitney test was used for the pairwise comparisons. ****p < 0.0001. **B** Seroprevalence of anti-al34k2 and anti-alSGE IgG antibodies. Error bars indicate 95% confidence intervals

We found that IgG responses to al34k2 were significantly higher in exposed subjects from Réunion Island than in the unexposed control group (*p* < 0.0001, Fig. 1A), a result that is fully consistent with the difference in anti-alSGE IgG antibody levels previously measured in the same cohort of individuals by Doucoure and collaborators [20] (*p* < 0.0001, Fig. 1A). The remarkably higher anti-al34k2 IgG antibody levels in exposed subjects points to al34k2 as a highly valuable antigen to reveal human exposure to bites of the tiger mosquito *Ae. albopictus*, especially considering the characteristics of the study area and previous experimental evidence. First, sera were collected during the high mosquito density season in an area where *Ae. albopictus* is the only *Aedes* species known to bite humans, which facilitates result interpretation. Second, within the limits of a comparison between ELISA experiments performed independently and in different conditions, IgG antibody levels and seroprevalence to al34k2 (0.67 ± 0.089) were in good agreement with IgG responses and prevalence to alSGE (0.87 ± 0.063; Fig. 1B). The higher IgG responses and prevalence to alSGE, as well as the relatively low Spearman correlation coefficient between IgG responses to the two antigens (*r* = 0.50, 95% CI 0.34 to 0.63, *p* < 0.0001), should not be too surprising considering that (1) *Aedes* saliva is a complex mixture of 100–150 proteins [23] and (2) human immune response to mosquito salivary proteins is both quantitatively and qualitatively heterogeneous [49]. Notably, seroprevalence values to al34k2 (66.7%) and alSGE (78.0%) suggest that recombinant al34k2 can detect > 85% of individuals seropositive to alSGE, indicating that al34k2 may be a convenient and reliable proxy for whole saliva or SGE as an indicator of human exposure to the tiger mosquito.

### IgG responses to al34k2 in subjects from Bolivia naturally exposed to *Aedes aegypti*

The 34k2 salivary proteins from *Ae. albopictus* and *Ae. aegypti* share a relatively high amino acid identity (62%), and understanding the degree of species-specificity of human anti-al34k2 IgG responses represents a question of interest. This is especially relevant considering that in a murine model the 34k2 proteins from *Ae. albopictus* (al34k2) and *Ae. aegypti* (ae34k2) were both immunogenic but, surprisingly, there was no cross-reactivity between these two antigens. In fact, sera of mice immunized to *Ae. albopictus* saliva did not include IgG antibodies recognizing the ae34k2 protein; vice versa, mice exposed to *Ae. aegypti* did not carry IgG antibodies able to recognize al34k2 [45]. The analyses of human cohorts from Northeast Italy [46] and from Réunion Island (see above), which refer to subjects naturally exposed to bites of *Ae. albopictus*, provided no clues on the potential usefulness of the al34k2 protein to reveal human exposure to *Ae. aegypti*. To gain some insights into this issue we analyzed IgG responses to al34k2 in a group of individuals from Bolivia who were exposed to bites of *Ae. aegypti* but not of *Ae. albopictus*. Notably, these samples represent a subset of sera from a larger study designed to evaluate specific IgG antibody responses to *Ae. aegypti* salivary gland extracts (aeSGE) [21]. Therefore, this Bolivian cohort appeared to be a perfect complement of the Réunion Island cohort described before, because of both the specificity of exposure to *Ae. aegypti* and the availability of data on IgG responses to aeSGE.

**Fig. 2:**
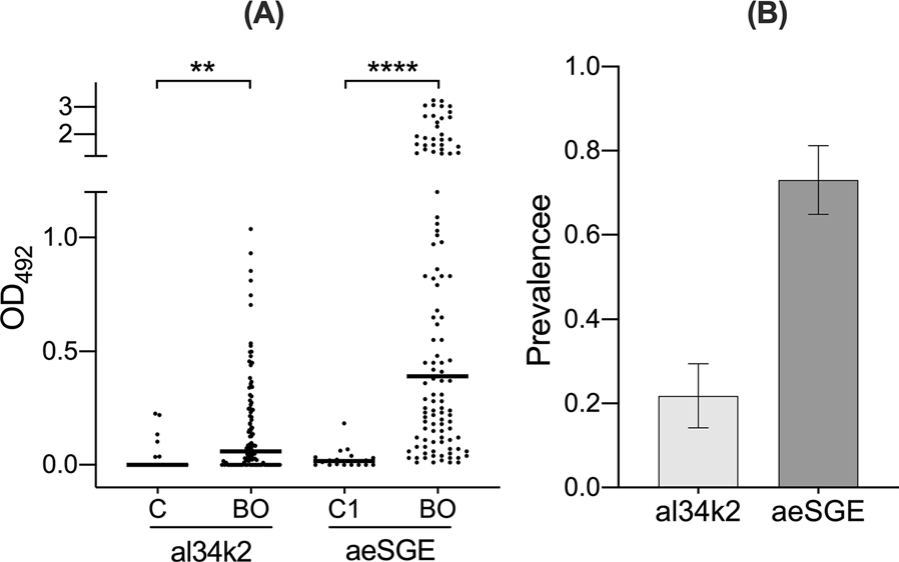
IgG responses to al34k2 in individuals from Bolivia and unexposed controls. **A** Scatter plot of IgG responses to al34k2 in subjects from Bolivia (BO, *n* = 115) and in unexposed controls (C, *n* = 20). IgG responses to aeSGE in the same individuals (BO, *n* = 115) and in unexposed controls (C1 *n* = 18), as previously determined (21), are shown for visual comparison. Horizontal bars indicate median values. al34k2, *Ae. albopictus* 34k2 recombinant protein; aeSGE, *Ae. aegypti* salivary gland extracts. IgG responses are expressed as OD values. The non-parametric Mann-Whitney test was used for the pairwise comparisons. **p < 0.01; ****p < 0.0001. **B** Seroprevalence of anti-al34k2 and anti-aeSGE IgG antibodies. Error bars indicate 95% confidence intervals

Interestingly, anti-al34k2 IgG levels were higher in sera of individuals from Bolivia than in the unexposed cohort (*p* = 0.0014, Fig. 2A). These same subjects, as previously determined by Doucoure and collaborators [21], showed significantly higher IgG responses to *Ae. aegypti* salivary gland extracts compared to unexposed controls (*p* < 0.0001). These observations suggest that individuals exposed to *Ae. aegypti* develop IgG antibodies against ae34k2 which can recognize, at least to a certain extent, the *Ae. albopictus* 34k2 protein. However, this cross-recognition appeared rather weak, as suggested by the low median anti-al34k2 IgG value (below the cut-off level) and by the low prevalence value (0.22 ± 0.038) compared to aeSGE (0.73 ± 0.042; Fig. 2B). Overall, these observations suggest that the al34k2 antigen from *Ae. albopictus* may be able to detect exposure to *Ae. aegypti*; however, sensitivity may be too low as indicated by the fact that recombinant al34k2 can only detect around 30% of individuals seropositive to aeSGE.

### IgG responses to ae34k2 in subjects from Bolivia, Réunion Island and in unexposed controls

**Fig. 3:**
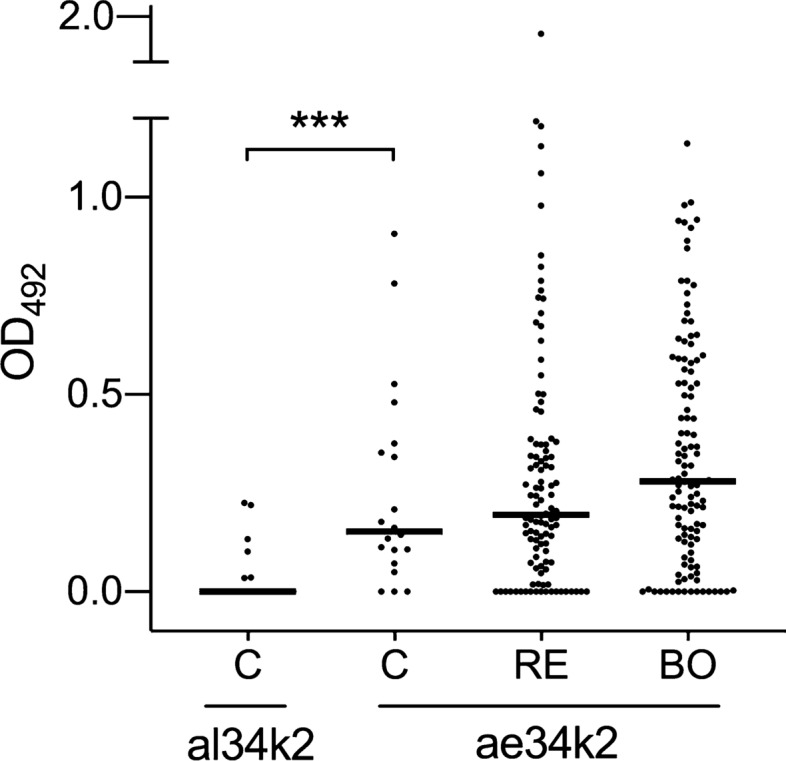
IgG responses to ae34k2 in individuals from Réunion Island, Bolivia and in unexposed controls. Scatter plot of IgG responses to ae34k2 in unexposed controls (C, *n* = 20), in subjects from Réunion Island (RE, *n* = 108) and from Bolivia (BO, *n* = 115). IgG responses to al34k2 of unexposed controls (C, *n* = 20) are shown for comparison. Horizontal bars indicate median values. al34k2, *Ae. albopictus* 34k2 recombinant protein; ae34k2, *Ae. aegypti* 34k2 recombinant protein. IgG responses are expressed as OD values. The non-parametric Wilcoxon matched pair signed rank test was used for the pairwise comparisons in unexposed controls. ***p < 0.001

The two cohorts from Réunion Island and Bolivia, due to their peculiarities, also represented an ideal sample to evaluate IgG responses to the *Ae. aegypti* 34k2 salivary protein, which we had previously shown to be immunogenic to mice [45]. Surprisingly, anti-ae34k2 IgG levels were significantly higher than anti-al34k2 IgG levels in sera of unexposed individuals (*p* = 0.0003, Wilcoxon matched-pair test) but not significantly different between unexposed and exposed, from either Réunion Island or Bolivia (*p* > 0.05, Mann–Whitney test; Fig. 3). The different response of the unexposed control group to al34k2 (mean 0.037, median 0.000) and ae34k2 (mean 0.252, median 0.153) suggests a high background level with the ae34k2 antigen, something that we did not observe in previous experiments in mice [45]. We do not know the reason for this high background observed with human sera, possibly due to cross-reactivity to some unknown antigen(s). However, note that the two recombinant proteins, which were expressed in *E. coli* and purified with essentially identical experimental procedures [45], had only minimal and similar traces of endotoxin, as quantified by the Limulus Amebocyte Lysate assay (Pierce 88,282). According to these observations, the use of the *Ae. aegypti* ae34k2 salivary protein, or at least of this recombinant version, does not appear suitable for the development of immunoassays to evaluate human exposure to *Aedes* mosquitoes.

## Conclusions

In conclusion, this study consolidates previous observations made in temperate areas of Northeast Italy [46] and clearly establishes the reliability and specificity of anti-al34k2 IgG responses to evaluate human exposure to *Ae. albopictus*. Importantly, the results obtained in Réunion Island extend the possible use of the al34k2 antigen to tropical and subtropical areas, where dengue or chikungunya transmissions are endemic and arboviral circulation is maintained by the tiger mosquito. This opens new scenarios for the development of protocols that may complement classical entomological measures. In fact, the serological evaluation of human exposure to *Ae. albopictus* may allow to improve monitoring and control of vector populations and possibly provide novel tools for arboviral transmission risk assessment. In particular, IgG responses to al34k2 may help to estimate efficacy of interventions against the tiger mosquito, from classical insecticide-based control of larval and adult stages to more environmentally friendly alternatives as the sterile insect technique (SIT), which is actually under implementation in Réunion Island [50]. The ability of the al34k2 antigen to reveal, at least in part, also exposure to *Ae. aegypti* suggests its possible use even in areas where both vectors are present. However, considering the low level of cross-reactivity, a possible perspective may be the development of immunoassays based on a combination of the Nterm-34 kDa peptide, which is designed on ae34k2, and of the al34k2 protein. This may provide an assay capable to reveal with higher sensitivity human exposure to both *Ae. aegypti* and *Ae. albopictus*. Overall, the addition to our portfolio of novel mosquito salivary antigens suitable for the evaluation of human-vector contact is expected to significantly enrich our toolbox for the control of mosquito-borne diseases. Such complementary tools, which can be employed for epidemiological studies, and possibly for the evaluation of transmission risk, may be especially useful when implementation of classical entomological methods is challenging (low vector density, logistic constraints, limited resources, etc.) or when the simultaneous determination of exposure to vector and to specific circulating pathogen(s) by serological measurements may be needed.

## Data Availability

Data generated during this study are included in this published article (and its supplementary information files). The datasets used and/or analysed during the current study are available from the corresponding author on reasonable request.
